# A SCOPING REVIEW OF OUTCOME AND OUTCOME MEASURE SETS FOR ADULTS WITH SPINAL CORD INJURY, STROKE, AND TRAUMATIC BRAIN INJURY

**DOI:** 10.2340/jrm.v58.45634

**Published:** 2026-07-17

**Authors:** Snigdha WIDGE, Ahlam ZIDAN, Alyson KWOK, Mahtab AZHDAR, Sarah KALIS, Liz DENNETT, Erin MCCABE

**Affiliations:** 1 Faculty of Rehabilitation Medicine, University of Alberta, Edmonton; 2 Geoffrey and Robyn Sperber Health Sciences Library, University of Alberta, Edmonton, Canada

**Keywords:** learning health system, neurorehabilitation, core outcome sets, outcome measures, spinal cord injury, stroke, traumatic brain injury

## Abstract

**Background:**

Standardized outcome measurement is essential for embedding neurorehabilitation within learning health systems. However, existing core outcome sets and standard outcome measure sets for stroke, traumatic brain injury, and spinal cord injury have not been synthesized across conditions.

**Objective:**

To synthesize outcome domains and measures included in core outcome sets and standard outcome measure sets for stroke, traumatic brain injury, and spinal cord injury, and examine involvement of persons with lived experience in their development.

**Methods:**

A scoping review was conducted following JBI methodology and PRISMA-ScR guidelines. MEDLINE, Embase, CINAHL, and PsycINFO were searched (May 2025). Studies describing development or evaluation of core outcome sets or standard outcome measure sets for adults with stroke, traumatic brain injury, or spinal cord injury were included. Outcomes and measures were mapped to the International Classification of Functioning, Disability and to “meaningful outcomes”.

**Results:**

Forty-eight studies were included. Over half (54%) of meaningful outcomes were shared across all 3 conditions; however, only 3% of outcome measures were recommended across conditions. Measures were absent for some patient-prioritized domains, including accessible environments and attitudes of others. Persons with lived experience were involved in 35% of studies.

**Conclusion:**

While rehabilitation priorities are conceptually aligned across conditions, measurement remains fragmented and patient engagement is limited. Harmonized, patient-informed, and implementable outcome strategies are needed to support learning health systems-driven neurorehabilitation.

## LAY ABSTRACT

Rehabilitation helps people recover after stroke, spinal cord injury, or traumatic brain injury. To understand whether rehabilitation is working, health professionals measure “outcomes”, which are the things that matter in recovery, such as walking ability, communication, independence in daily activities, pain, and participation in work or community life. They use different tools or questionnaires to track this progress. In this review, we examined recommended lists of outcomes and measurement tools for these 3 conditions. We found that many outcomes are similar across conditions. However, different tools are often used to measure the same outcomes, which makes it difficult to compare results across programmes and improve care. We also found that people with lived experience were not always involved in deciding what outcomes should be measured. Our findings highlight the need for a shared set of outcome measures, informed by patients and clinicians, to strengthen neurorehabilitation practice and research.

Neurorehabilitation services can be highly effective in supporting recovery and reducing disability after acquired neurological injuries (1–3). However, individuals with neurological injuries (i.e., stroke, traumatic brain injury [TBI], spinal cord injury [SCI]) vary in their responses to rehabilitation interventions, making it difficult to predict who would benefit from specific interventions and at what stage of recovery (4). As a result, many patients do not receive rehabilitation tailored to their individual needs, potentially leading to suboptimal outcomes and decreasing the efficiency of rehabilitation services (5).

Learning health systems (LHS) are an approach to developing and using precision rehabilitation (5). LHS leverage heterogeneous data from routine clinical practice, analyse the data to understand variability in recovery patterns, then rapidly translate that knowledge back into clinical practice (6, 7). A critical component of a LHS is the standardized measurement of patient outcomes across a continuum of care (8), requiring a standardized approach to using patient-reported outcome measures, which assess patient perspectives on their health and well-being through standardized questionnaires (9), as well as clinical outcome measures, which evaluate impairment and function through clinician observation (10).

To embed standardized measurement within learning health systems, it is first necessary to agree on a common set of key outcomes and the specific tools used to measure them. Core outcome sets (COS) are standardized collections of the minimum outcomes that should be measured and reported in all clinical studies for a specific health condition, whereas standard outcome measure sets (SOMS) specify the instruments or tools used to assess those outcomes (11). COS/SOMS development generally involves 3 steps: (*i*) a literature review to map existing outcomes or measures, (*ii*) a consensus process (e.g., Delphi rounds and a final meeting), and (*iii*) publication and registration to support uptake (12–14).

Existing COS and SOMS for stroke (15–20), TBI (21, 22), and SCI (23–27) are often designed for distinct stages of recovery (e.g., acute care, rehabilitation, long-term monitoring) or a specific domain or impairment (e.g., mobility, balance, swallowing). Furthermore, these sets are not integrated across stroke, TBI, and SCI, which means that neurorehabilitation practitioners must navigate a complex outcome measure landscape with multiple different measures recommended for the same conceptual domain (e.g., different tests of walking speed), even when patients share common functional goals and patient care pathways (i.e., acute care to rehabilitation to community). Major data standardization initiatives, such as the National Institute of Neurological Disorders and Stroke Common Data Elements guidelines, have begun to recognize this complexity (28). Because these guidelines were developed as separate catalogues for stroke, TBI, and SCI, they are highly effective for tracking condition-specific outcomes. However, as the field shifts towards integrated care models, bridging these separate catalogues has become a recognized priority. Efforts like the recent NeuroRehab Common Data Elements initiative, as well as the ClinFIT (a universal functioning information tool based on the ICF) (29), highlight growing momentum to harmonize outcome measurements across conditions, yet translating these top-down administrative standards into a unified clinical workflow across different populations remains an ongoing challenge (30). Synthesizing outcomes and outcome measures across existing COS and SOMS in these populations provides a foundation to develop a standardized approach to outcome measurement within a neurorehabilitation LHS. To our knowledge, no prior review has synthesized core outcome sets and standard outcome measure sets across stroke, traumatic brain injury, and spinal cord injury to examine cross-condition alignment.

Therefore, the purpose of this scoping review is to synthesize existing COS and SOMS for people who have experienced a stroke, TBI, or SCI. Specifically, this review aimed to: (*i*) describe outcome domains included in COS for these populations and synthesize them across populations, (*ii*) identify outcome measures included in SOMS for these populations and synthesize them across conditions, and (*iii*) examine the involvement of persons with lived experience (PWLE) in the development of these sets.

## METHODS

### Protocol and registration

We followed the methodology outlined by the Joanna Briggs Institute (JBI) for conducting scoping reviews (31) and this review is reported in accordance with the PRISMA Extension for Scoping Reviews (PRISMA-ScR) checklist (32). A protocol was developed *a priori* and registered on Open Science Framework (https://doi.org/10.17605/OSF.IO/ZS48Y). The scoping review methodology is appropriate because it allows a systematic mapping of the full breadth of COS and SOMS, clarifying what has been studied, how it has been studied, and where evidence gaps remain.

### Eligibility criteria

We included studies that focused on sets for adults aged 18 years or older who had experienced either stroke and/or traumatic brain injury (TBI) and/or spinal cord injury (SCI), in any clinical or research setting, including acute care, rehabilitation, primary care, long-term monitoring, and for any rehabilitation discipline (e.g., physiotherapy, speech/language pathology, occupational therapy, etc.). We also included sets for adults with general neurological conditions, if they covered at least 1 of the populations identified above.

We included all sources describing the development or an evaluation of a set. We included studies describing the development or evaluation of a set to capture both the recommended outcomes/measures and details of the consensus processes, including PWLE involvement. For SOMS, we included any set that has at least 1 clinical outcome measure, including patient-reported outcome measures (PROMs), clinician-reported outcome measures, or performance-based measures (i.e., where the patient performs a set of movements or tasks and is scored based on time to complete a task, distance, or a qualitative assessment of movement quality [33]). We only included sources that described a transparent consensus process for the development of the COS/SOMS or conducted an expert evaluation of measurement properties of outcome measures for SOMS.

Studies were excluded if they focused exclusively on paediatric populations (< 18 years) or addressed only mild brain injury or concussion, as rehabilitation care pathways for this population are different.

### Search strategy

We conducted comprehensive searches of MEDLINE, Embase, CINAHL, and PsycINFO without imposing any search limits. A health sciences librarian designed the search strategy, and the final searches were executed on 27 May 2025. The reference lists of included studies were hand-searched by a single reviewer to identify additional relevant studies that may not have been captured through the database searches. The full strategy for each database is included in Appendix SI.

### Selection of sources of evidence

All retrieved citations were imported into Covidence, and duplicates were removed. Titles and abstracts were screened independently by SW, MA, and SK against the inclusion criteria. Inter-rater agreement for title and abstract screening ranged from 59.4% to 83.3%. The full text of selected citations was then assessed independently by SW and MA. Reference lists of included studies were hand-searched by SW to identify additional relevant studies. Reasons for excluding sources at the full-text stage were documented. Regular meetings were held to discuss discrepancies and support inter-rater consistency. Any unresolved discrepancies were resolved by EM following team discussion. Study screening and selection were conducted using Covidence. Data charting, synthesis, and mapping were completed using Microsoft Excel (Microsoft Corp, Redmond, WA, USA). No artificial intelligence (AI) tools, including AI-assisted features available within Covidence, were used during the review process.

### Data charting process

A draft charting form was piloted and refined by SW and MA. Using the final template, SW and MA independently extracted data from 10 studies, discrepancies were resolved through team discussion. SW continued with the remaining studies. The data extraction form included study characteristics, target population, consensus processes, concepts of interest, settings, involvement of people with lived experience, identified outcomes, measures, and measurement properties described in the study.

### Synthesis of data

We calculated descriptive statistics to describe the characteristics of the included articles. We then built 3 matrices which collated the identified COS and SOMS. The first described each outcome from COS, its definition, and the number of sources recommending that domain. For the second matrix, we synthesized each outcome measure identified in the SOMS by target population(s), construct(s), context(s) of use, and the International Classification of Functioning, Disability and Health (ICF) (34) domain it covers, along with the number of sources recommending that measure and the psychometric properties reported in the studies. The third matrix included COS by ICF domains.

### ICF mapping

We mapped each identified outcome, outcome measure, and ICF domain to ICF chapters using a standardized protocol. EM and SW independently examined outcome definitions, outcome measure manuals, and individual items. Inter-rater reliability for ICF coding was assessed using Cohen’s kappa (κ = 0.82). The reviewers then extracted key concepts to identify the ICF chapters covered by each outcome or measure. A 25% subsample was used for calibration, after which reviewers met to discuss and resolve discrepancies. The reviewers proceeded to code the rest independently; discrepancies were resolved through consensus of the research team.

### Meaningful outcomes

After the ICF mapping, we found that ICF Chapters were too broad to be meaningfully interpreted (e.g., mental functions include cognition and mental health, which are important concepts to separate), while the ICF Domains were too granular to allow meaningful interpretation across conditions. Therefore, we developed a set of “meaningful outcomes” by combining outcomes and ICF domains into broader categories. This approach was adopted to balance conceptual specificity and cross-condition interpretability, addressing limitations of both high-level ICF chapters and highly granular ICF domains. The development of the meaningful outcome categories was led by SW, AK, AZ, and EM through an iterative inductive coding process and consensus discussions. We used an inductive approach to develop meaningful outcomes. First, we independently reviewed the outcomes identified from the COS and the ICF domains and created codes representing similar concepts across outcomes and domains. The team then met to compare and discuss their codes. Through an iterative consensus process, related codes were combined resulting in a final set of meaningful outcomes and accompanying definitions, providing a pragmatic middle ground for synthesis and interpretation. The meaningful outcomes are more concrete and interpretable for people with lived experience, clinicians, and other interest-holders.

## RESULTS

### Selection of reviews

**Fig 1:**
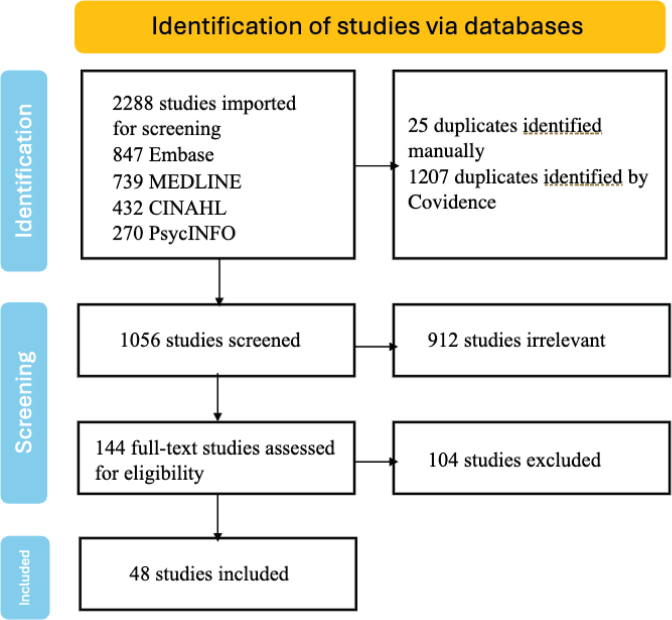
PRISMA flowchart.

The PRISMA flow diagram of study selection is presented in Fig. 1. Search of electronic databases yielded 2,288 records. After removing 1,232 duplicates, 1,056 titles/abstracts were screened. In total, 144 full texts were assessed for eligibility, and 48 studies met the inclusion criteria and were retained in the review.

### Characteristics of included studies and sets

The studies were published from 2004 to 2024, and a description of their characteristics is provided in Appendix SII. Studies encompassed 18 COS, 26 SOMS, and 4 with both COS and SOMS across conditions. Stroke was the target population in 30 studies, TBI in 7 studies, and SCI in 6 studies. Additionally, 5 studies focused on broader neurological populations. Some 54% of the studies had an international scope, while 27% were national; 44 studies reported on the development of COS or SOMS and 4 studies evaluated validation of existing sets. Earlier studies (2004–2010) were primarily developed for acute or inpatient rehabilitation settings, while studies published after 2015 more frequently included community-based or cross-continuum settings. The most common consensus development method was the Delphi or modified Delphi approach, used in 52% of the studies. Iterative or multi-step processes and other consensus-based approaches were each used in 6 studies (13% each). Surveys were used in 3 studies (6%), while 3 studies (6%) did not specify a particular consensus method. Less frequently used approaches included feedback-based processes (4%) and nominal group techniques (2%), meetings (2%), and focus groups (2%). The ICF was used as a central framework in 21% of the studies. Seven studies reported on the development of ICF Core Sets, and 3 studies reported on their validation. These included ICF Core Sets for stroke (*n* = 4), traumatic brain injury (*n* = 2), spinal cord injury (*n* = 3), and other neurological conditions (*n* = 1). People with lived experience (PWLE) were involved in 35% of the included studies. In 6% of studies, PWLE participated in focus groups, surveys, or interviews to identify outcomes important to them but were not involved in the consensus process. In another 6% of studies, PWLE were the only interest holders involved in decision-making during the consensus process.

### Outcomes

From the studies developing a COS (not using ICF), a total of 137 outcomes were recommended across the included studies: 106 for stroke, 20 for SCI, and 12 for Blunt Cerebrovascular Injury (BCVI) (see Appendix SIII). The outcome, quality of life, mapped to the ICF chapter Community, Social and Civic Life, was the only outcome recommended for more than 1 population. It was also the most frequently reported outcome overall (*n* = 8).

### ICF categories

For 7 studies on ICF Core Set development and 3 on validation, a total of 298 domains were identified across all included sources (see Appendix SIV). Altogether, 183 domains were identified for stroke, 208 for SCI, 138 for TBI, and 79 for general neurological conditions. In the Body Structures category (*n* = 28 domains), with structures related to the nervous system (25%) and structures related to movement (57%) being the most common. For Body Functions (*n* = 104 domains), neuromusculoskeletal and movement-related functions (27%) were most common, followed by sensory functions and pain (19%). In the Activities and Participation category, 101 domains were recommended, with mobility being the most common (38%).

**Fig. 2:**
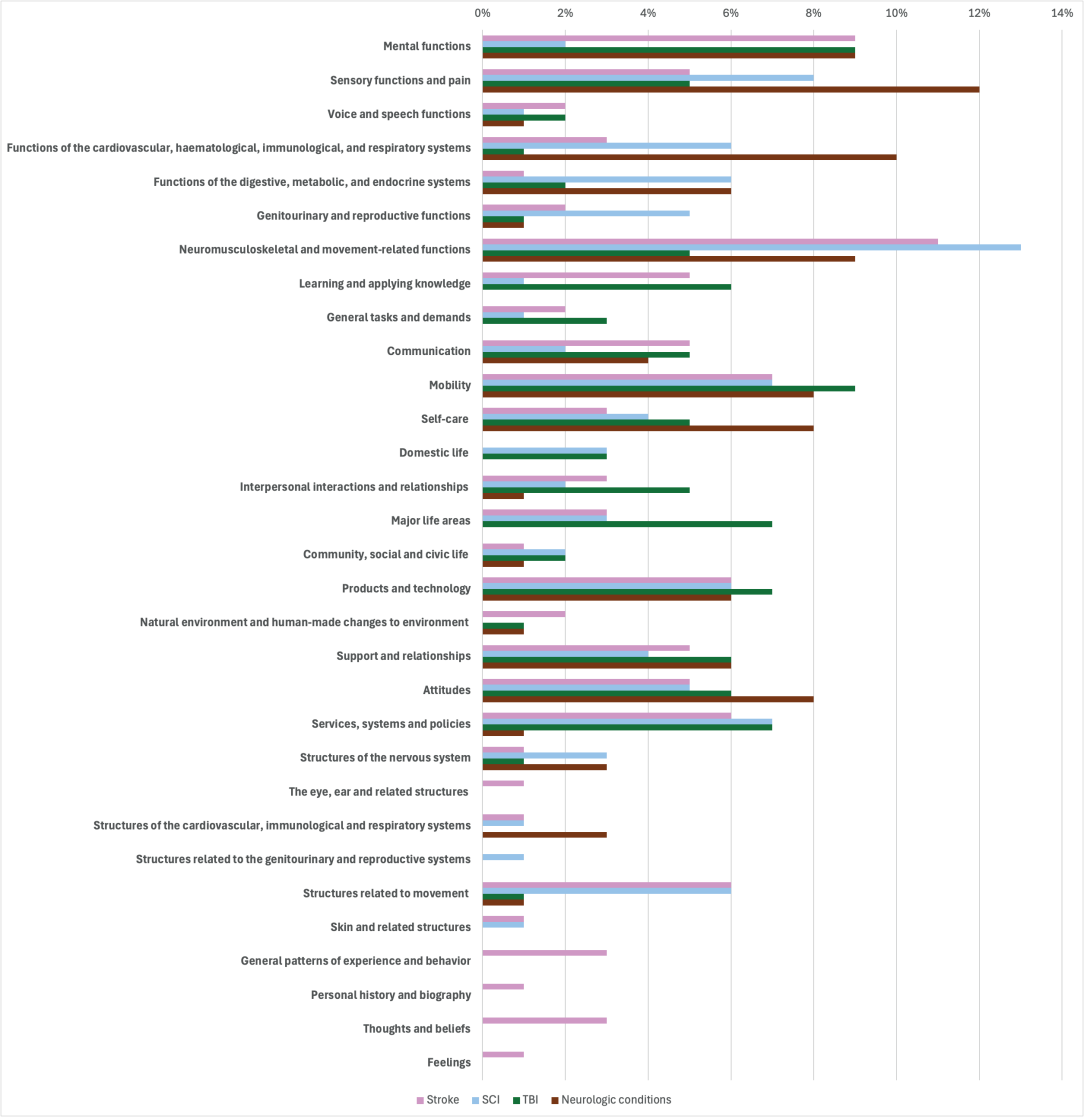
Proportion of ICF Core Sets represented in each ICF chapter by population (*n* = 10 studies).

Fewer environmental factors were included (*n* = 52), but products and technology (29%) and services, systems, and policies (27%) were most common. Very few personal factors were identified (*n* = 13). Fig. 2 illustrates the differing emphasis of ICF chapters across populations.

### Meaningful outcomes

**Fig. 3:**
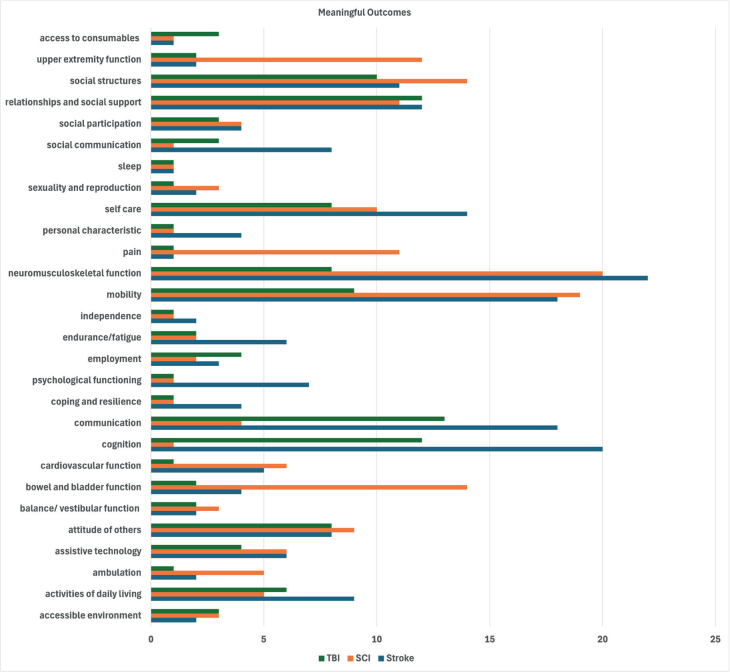
Number of COS that included the 28 most common meaningful outcomes, by condition.

We identified 50 meaningful outcomes across the COS (ICF and non ICF) (see Appendix SV for terms and operational definitions). A subset of meaningful outcomes (54%) was present across all 3 conditions, such as neuromusculoskeletal function, mobility, relationships and social support, cognition and self-care (Fig. 3).Some meaningful outcomes were condition-specific. For stroke, uniquely reported outcomes included falls incidence (included in 6 COS), knowledge and beliefs related to falling (included in 7 COS), and quality of life (5 COS). For spinal cord injury, condition-specific outcomes included blood pressure (3 COS), caregiver burden (1 COS), education (4 COS), and housing (1 COS).

### Outcome measures

A total of 275 outcome measures were recommended across the included studies: 130 for stroke, 82 for spinal cord injury (SCI), and 93 for traumatic brain injury (TBI) (Appendix SVI).

Multiple measures were used to assess the same meaningful outcomes. No measures were identified for the following meaningful outcomes: access to consumables, social structures, social communication, attitudes of others, and accessible environment.

**Fig. 4:**
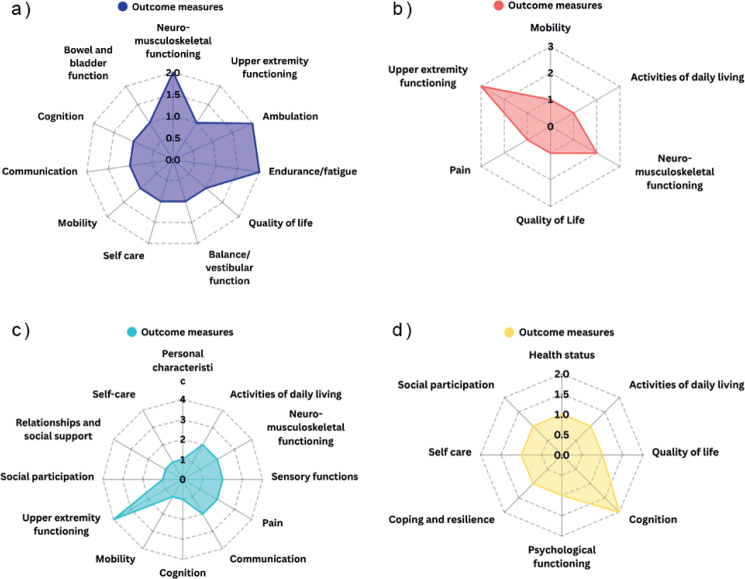
Representation of the most frequently recommended outcome measures to the meaningful outcomes they assess. (A) Most common recommended measures across all 3 conditions. (B) Most common outcome measures recommended for SCI. (C) Most common outcome measures recommended for stroke. (D) Most common outcome measures recommended for TBI.

A small number of outcome measures (3%) were recommended across all 3 conditions. These included the Action Research Arm Test (ARAT) (*n* = 6), the 10-Metre Walk Test (10MWT) (*n* = 10), the 6-Minute Walk Test (6MWT) (*n* = 9), the EQ-5D (*n* = 10), the Berg Balance Scale (BBS) (*n* = 10), the Functional Independence Measure (FIM) (*n* = 9), and the Ashworth Scale and Modified Ashworth Scale (*n* = 5), were also recommended across all conditions (Fig. 4A).

For stroke, the frequently recommended measures were the Fugl-Meyer Assessment (FMA) (*n* = 10), National Institutes of Health Stroke Scale (NIHSS) (*n* = 4), Box and Block Test (*n* = 4), Montreal Cognitive Assessment (*n* = 3), hand dynamometry (*n* = 3), Nine-Hole Peg Test (*n* = 3), Reintegration to Normal Living Index (RNLI) (*n* = 3), and Motor Activity Log (*n* = 3). (Fig. 4B).

For SCI, the frequently recommended measures were the 36-Item Short-Form Health Survey (SF-36) (*n* = 3), Spinal Cord Independence Measure (SCIM) (*n* = 3), Wheelchair Skills Test (WST) (*n* = 2), Sollerman Hand Function Test (*n* = 2), Graded Redefined Assessment of Strength, Sensibility and Prehension (GRASSP) (*n* = 2), Capabilities of Upper Extremity (CUE) (*n* = 2), Penn Spasm Frequency Scale (*n* = 2), Classification System for Chronic Pain (*n* = 2), and Spinal Cord Injury Hand-Held Myometer (*n* = 2) (Fig. 4C).

For TBI, the frequently recommended measures were the Glasgow Coma Scale (GCS) (*n* = 3), Trail Making Test (TMT) (*n* = 3), Mayo–Portland Adaptability Inventory (MPAI) (*n* = 3), Rey Auditory Verbal Learning Test (RAVLT) (*n* = 2), Quality of Life after Brain Injury scale (QOLIBRI) (*n* = 2), and Sydney Psychosocial Reintegration Scale (SPRS) (*n* = 2) (Fig. 4D).

## DISCUSSION

This scoping review identified outcomes and outcomes measures included in COS and SOMS for stroke, SCI, and TBI examined commonalities across populations, and described the involvement of persons with lived experience in their development.

### Consistency in recommendations across all 3 conditions

Our findings reveal that although stroke, SCI, and TBI share many common rehabilitation priorities in terms of outcomes, approaches to measuring outcomes remain largely condition-specific and poorly harmonized. There was substantial overlap in meaningful outcomes across conditions, with more than 50% of outcomes shared between stroke, SCI, and TBI. In contrast, only 3% of outcome measures were recommended across all 3 conditions, indicating a marked gap between conceptual alignment (i.e., what should be measured) and measurement alignment (i.e., how it is measured). This reflects a broader pattern within clinical research in which new condition-specific measures are developed rather than validating or adapting existing instruments in the new population (9, 35).

These acute-onset neurological conditions often result in many shared functional, psychosocial, and participation-related challenges, suggesting that many outcome priorities would be common across populations. However, our findings indicate that different condition-specific measures are used to assess these outcomes. While condition-specific measures may be appropriate in some contexts, it is not always necessary; common measures are often used to capture similar constructs across populations. The Patient-Reported Outcomes Measurement Information System initiative was founded on this premise, developing standardized, cross-condition measures of core health domains to improve comparability of outcomes across patient conditions and reduce redundancy in measure development (36). Similar harmonization efforts could be extended beyond patient-reported outcomes to clinician-reported and performance-based measures in neurorehabilitation.

It should be noted that interpretation of degree of overlap is influenced by the composition of the literature, which was dominated by stroke studies. Because COS and SOMS have been more extensively developed for stroke than for TBI or SCI, the observed overlap in outcomes likely underestimates the true commonality in outcomes across these conditions. A similar pattern may apply to outcome measures, meaning the estimate of 3% of outcome measures may in fact be slightly higher in practice.

### Integration into routine practice

The integration of standardized outcome measures into routine clinical practice is important to generating outcomes data necessary for a learning health system (8, 37). However, among the included studies, only 4 focused on validation or evaluation of a COS or SOMS, and none explicitly evaluated the implementation of COS or SOMS within real-world clinical settings.

Implementing outcome measurement is complex and often requires changes to clinician workflows, documentation practices, data infrastructure, and institutional policies, which can create substantial barriers to integration (38–40). Without explicit consideration of feasibility, burden, and interpretability, consensus-based outcome sets may be difficult to operationalize in practice. For outcome measurement to meaningfully support system improvement, selected measures must be not only valid and relevant but also practical, sustainable, and suitable for the intended context of use.

Embedding harmonized measures within routine care aligns with learning health system principles, which emphasize the continuous use of outcome data to inform clinical decision-making and quality improvement. In neurorehabilitation, clinicians frequently treat stroke, SCI, and TBI populations within the same practice environment, yet traditional patient care pathways remain separated by diagnosis. Using common measures across conditions would simplify outcome data collection processes in neurorehabilitation, facilitate more consistent benchmarking across populations, and reduce clinician administrative burden of outcome measures, all of which enhance likelihood of uptake by clinicians and sustainability of a standardized approach to outcome measurement (41–43).

Importantly, establishing a common measurement framework would allow for the direct comparison of clinical outcomes across historically siloed populations. These comparative data provide a lens to distinguish between condition-specific rehabilitation trajectories and transdiagnostic functional presentations (e.g., shared profiles of cognitive fatigue and spatial neglect). For health system administrators, shifting from diagnosis-driven to presentation-driven models of care would allow for the design of patient care pathways that are more tailored to an individual’s true functional needs. Essentially, using a common measure set would allow the development of the empirical foundation necessary to allocate rehabilitation resources more accurately and equitably based on functional severity rather than ICD-10 codes.

### Involvement of PWLE

The limited engagement of PWLE in consensus processes may contribute to gaps between formally recommended outcomes and those most valued by patients. Approximately one-third of studies (35%) reported the involvement of PWLE, and only 3 described PWLE as the sole contributors to the consensus process. In our review, measures were not identified for accessible environments, employment, social structures, or social communication, despite these meaningful outcomes being recommended for all 3 conditions. The absence of measures in these domains suggests a structural gap between patient-prioritized outcomes and measurement development, which may reflect the limited involvement of PWLE in outcome measure set development or the possibility that suitable instruments have not yet been developed. More broadly, qualitative literature suggests that patients often emphasize outcomes such as emotional and psychological well-being, accessible environments, personal characteristics (e.g., self-acceptance, self-esteem), relationships and social support, coping and resilience, independence, social participation, social structures, social communication, and employment (44–46). Though these types of outcomes were represented in some of the COS, they were largely absent from the SOMS.

The International Consortium for Health Outcomes Measurement and Outcome Measures in Rheumatology initiatives recommend the involvement of PWLE in set development (47, 48). Recognizing a need for more guidance in this area, the COMET initiative created a guide to Patient and Public Involvement in COS development (47, 48). However, there is limited guidance on what would be considered meaningful involvement and how to weight the priorities of PWLE against those of other interest-holders (i.e., clinicians, researchers, health system administrators) (49). Centring PWLE within COS/SOMS development aligns with broader trends toward co-design in health research and is consistent with many LHS frameworks which centre patients and families in LHS processes (50–54).

### Limitations

This scoping review followed established methodologies, using a comprehensive search strategy and rigorous analytic approaches. We extracted and reported detailed information on outcome set development methods (e.g., use of Delphi processes, consensus meetings, and stakeholder composition), allowing readers to consider findings within methodological variation of studies. Although we implemented a comprehensive search strategy, including multiple bibliographic databases, and hand-searching reference lists, some COS or SOMS may have been missed due to inconsistent terminology, grey literature reporting, or language restrictions. In addition, consistent with scoping review methodology, we did not formally assess the methodological quality of included COS or SOMS, and therefore findings should be interpreted as a mapping of existing recommendations rather than an appraisal of their rigour.

Mapping outcomes and measures to “meaningful outcomes” required interpretive judgement and may have introduced inconsistencies, particularly given variability in how outcomes were defined across studies. This was mitigated through independent coding by 2 reviewers and iterative consensus discussions. Finally, limited reporting on PWLE engagement restricted our ability to assess the depth and impact of their involvement, and patient contributions may therefore be underrepresented in our synthesis.

### Conclusion

This scoping review highlights both convergence and fragmentation within outcome measurement for stroke, SCI, and TBI. While there is substantial agreement on key rehabilitation outcomes across conditions, alignment in recommended measures remains limited, and meaningful involvement of people with lived experience is limited and inconsistently reported. Together, these findings suggest that future efforts should move beyond condition-specific recommendations toward harmonized, patient-informed, and implementable outcome measurement strategies for neurorehabilitation. Advancing cross-condition standardization, strengthening co-design with PWLE, and explicitly integrating feasibility considerations into COS and SOMS development will be critical to embedding outcome measurement within routine care and supporting learning health system goals in neurorehabilitation.

## Supplementary Material


